# Mesenchymal stem cells can modulate longitudinal changes in cortical thickness and its related cognitive decline in patients with multiple system atrophy

**DOI:** 10.3389/fnagi.2014.00118

**Published:** 2014-06-13

**Authors:** Mun Kyung Sunwoo, Hyuk Jin Yun, Sook K. Song, Ji Hyun Ham, Jin Yong Hong, Ji E. Lee, Hye S. Lee, Young H. Sohn, Jong-Min Lee, Phil Hyu Lee

**Affiliations:** ^1^Department of Neurology, Bundang Jesaeng General HospitalSeongnam, Korea; ^2^Department of Biomedical Engineering, Hanyang UniversitySeoul, South Korea; ^3^Department of Neurology, Jeju University College of MedicineJeju, Korea; ^4^Department of Neurology, Yonsei University College of MedicineSeoul, Korea; ^5^Department of Neurology, Wonju College of Medicine, Yonsei UniversityWonju, Korea; ^6^Department of Biostatistics, Yonsei University College of MedicineSeoul, Korea; ^7^Severance Biomedical Science Institute, Yonsei University College of Medicine SeoulKorea

**Keywords:** mesenchymal stem cells, multiple system atrophy, cortical thickness, cognition, clinical trial

## Abstract

Multiple system atrophy (MSA) is an adult-onset, sporadic neurodegenerative disease. Because the prognosis of MSA is fatal, neuroprotective or regenerative strategies may be invaluable in MSA treatment. Previously, we obtained clinical and imaging evidence that mesenchymal stem cell (MSC) treatment could have a neuroprotective role in MSA patients. In the present study, we evaluated the effects of MSC therapy on longitudinal changes in subcortical deep gray matter volumes and cortical thickness and their association with cognitive performance. Clinical and imaging data were obtained from our previous randomized trial of autologous MSC in MSA patients. During 1-year follow-up, we assessed longitudinal differences in automatic segmentation-based subcortical deep gray matter volumes and vertex-wise cortical thickness between placebo (*n* = 15) and MSC groups (*n* = 11). Next, we performed correlation analysis between the changes in cortical thickness and changes in the Korean version of the Montreal Cognitive Assessment (MoCA) scores and cognitive performance of each cognitive subdomain using a multiple, comparison correction. There were no significant differences in age at baseline, age at disease onset, gender ratio, disease duration, clinical severity, MoCA score, or education level between the groups. The automated subcortical volumetric analysis revealed that the changes in subcortical deep gray matter volumes of the caudate, putamen, and thalamus did not differ significantly between the groups. The areas of cortical thinning over time in the placebo group were more extensive, including the frontal, temporal, and parietal areas, whereas these areas in the MSC group were less extensive. Correlation analysis indicated that declines in MoCA scores and phonemic fluency during the follow-up period were significantly correlated with cortical thinning of the frontal and posterior temporal areas and anterior temporal areas in MSA patients, respectively. In contrast, no significant correlations were observed in the MSC group. These results suggest that MSC treatment in patients with MSA may modulate cortical thinning over time and related cognitive performance, inferring a future therapeutic candidate for cognitive disorders.

## INTRODUCTION

Multiple system atrophy (MSA) is a sporadic neurodegenerative disease characterized by α-synuclein-positive glial cytoplasmic inclusions (GCIs) with widespread neuronal loss in the basal ganglia, brainstem, cerebellum, and spinal cord. Clinically, the cardinal features of MSA include autonomic dysfunction, parkinsonism, cerebellar ataxia, and pyramidal signs in any combination, with autonomic dysfunction being an integral component (Stefanova et al., 2009). In addition, accumulating evidence suggests that along with cortical pathology involving frontal and temporal areas, cognitive function is more widely impaired in MSA than initially expected (Kawai et al., 2008; Chang et al., 2009).

Although the precise etiology and pathomechanisms of MSA remain unknown, several mediators including neuroinflammation, oxidative insult, mitochondrial dysfunction, and alterations in the blood–brain barrier have been suggested to play important roles in progression of MSA pathology (Shults et al., 2005; Stefanova et al., 2005; Ubhi et al., 2009; Song et al., 2011b). Many clinical trials focusing on modulation of MSA pathogenesis have been conducted to achieve neuroprotective properties; however, the results have been discouraging (Ubhi et al., 2011). In a randomized, double-blind, controlled trial of autologous mesenchymal stem cells (MSC), we obtained clinical and imaging evidence that MSC treatment could have a neuroprotective role in MSA patients (Lee et al., 2012b). In the present study, we evaluated the effects of MSC therapy on longitudinal changes in subcortical deep gray matter volumes and cortical thickness and their association with cognitive performance to elucidate whether MSC could modulate pathological processes in the cerebral cortex of MSA patients.

## MATERIALS AND METHODS

### SUBJECTS

Clinical and imaging data were obtained from our randomized trial of autologous MSC that was reported previously (Lee et al., 2012b). Briefly, patients with probable MSA-cerebellar type (Gilman et al., 1999) and baseline Unified Multiple System Atrophy Rating Scale (UMSARS, scores ranging from 0 to 106, with higher scores indicating greater neurological deficits) (Wenning et al., 2004) scores of 30–50 were enrolled in this study. Because functional deterioration in MSA is known to be more rapid in early stages than in advanced stages of the disease (Geser et al., 2006), we recruited MSA patients with mild-to-moderate stages of the disease with the expectation that early intervention would be more appropriate to achieve clinical efficacy. The subjects were randomly assigned to the MSC group receiving MSC (4 × 10^7^/injection) via intra-arterial and intravenous routes or placebo group. The detailed neuropsychological test and brain magnetic resonance imaging (MRI) were performed at baseline and at 12 months after first intra-arterial administration of MSC. All patients provided written informed consent. The trial was registered at ClinicalTrials.gov (ClinicalTrials.gov number NCT00911365), and the study protocol and consent form were approved by the Institutional Review Board for Human Investigation of Yonsei University Severance Hospital.

### IMAGE ACQUISITION

All MRI scans of patients were acquired using a Philips 3.0T scanner (Philips Intera, Philips Medical System, Best, The Netherlands) with a SENSE head coil (SENSE factor = 2). Head motion was minimized with restraining foam pads provided by the manufacturer. A high-resolution, T1-weighted MRI volume dataset was obtained using 3-dimensional T1-TFE sequence configured with the following acquisition parameters: axial acquisition with a 224 × 256 matrix; 256 × 256 reconstructed matrix with 182 slices; 220 mm field of view; 0.98 mm × 0.98 mm × 1.2 mm voxels; echo time 4.6 msec; repetition time 9.6 msec; flip angle 8°; slice gap 0 mm.

### CORTICAL THICKNESS ANALYSIS

Native MR images were registered into a standardized, stereotactic space using linear transformation (Collins et al., 1994). The N3 algorithm was used to correct images for intensity-based non-uniformities, resulting from inhomogeneity in the magnetic field (Sled et al., 1998). The non-brain tissues were removed from registered and corrected volumes (Smith, 2002) and then classified into white matter, gray matter, cerebrospinal fluid (CSF), and background using the Intensity-Normalized Stereotactic Environment for Classification of Tissues algorithm (Zijdenbos et al., 1996). The surfaces of the inner and outer cortices which consisted of 40,962 vertices were automatically extracted using the Constrained Laplacian-based Automated Segmentation with Proximities algorithm and inversely transformed to native space (MacDonald et al., 2000; Kim et al., 2005). In inner surface, a low-resolution, polyhedral surface deformed to fit the gray matter and white matter boundary and resampled to contain 81,920 polygons. Then, the outer cortical surface is expanded from inner surface using a Laplacian map defined between white matter and cerebrospinal fluid. The each vertex of inner and outer surfaces had the correspondence. Thus, the cortical thickness was defined as the Euclidean distance between linked vertices of the inner and outer native surfaces, because Lerch and Evans (2005) considered this method of cortical thickness measurement to be the simplest and most precise of several methods. The cortical thickness information was smoothed with 20 mm full-width half-maximum Gaussian smoothing kernel to increase the signal-to-noise ratio (Lerch and Evans, 2005; Im et al., 2006) and aligned to unbiased iterative surface template using vertex-wise, sphere-to-sphere, non-linear surface registration (Robbins et al., 2004; Lyttelton et al., 2007).

### SEGMENTATION AND VOLUMETRIC ANALYSIS FOR SUBCORTICAL STRUCTURE

Volume of subcortical structures was analyzed by using FMRIB’s Softward Library-FMRIB’s Integrated Registration and Segmentation Tool (FIRST, version 5.0). The method is the automated, model-based tool, which is optimized for registration and segmentation of deep subcortical structures including caudate, putamen, pallidum, and thalamus (Patenaude et al., 2011). The T1-weighted MR inputs are registered to Montreal Neurological Institute 152 standard space with 12 DOF (degree of freedom) affine transformation. Second, based on shape sample using another 12 DOF registration with application of subcortical mask, each structure was segmented. Then, boundary correction was performed with FSL’s FAST tool, which re-classifies boundary voxels to each subcortical structure in accordance with their intensity. Finally, summary of segmented volumes of bilateral caudate, pallidum and thalamus was acquired using routine FSL command: FSLstats (http://fsl.fmrib.ox.ac.uk/fsl/fslwiki/Fslutils).

### NEUROPSYCHOLOGICAL TESTS

To evaluate the changes in general cognition for follow-up period, the Korean version of the Montreal Cognitive Assessment (MoCA) was introduced in both groups of MSA patients. In addition, we used the Seoul Neuropsychological Screening Battery (SNSB) to determine the cognitive performance in both groups of patients (Kang and Na, 2003; Song et al., 2011a). The SNSB covers attention, language, praxis, visuoconstructive function, verbal and visual memory, and frontal/executive function. For these, the quantifiable tests comprised the digit span (forward and backward), Korean version of the Boston Naming Test, Rey Complex Figure Test (copying, immediate and 20-min delayed recall, and recognition), Seoul Verbal Learning Test (three learning-free recall trials of 12 words, 20-min delayed recall trial for these 12 items, and a recognition test), phonemic and semantic Controlled Oral Word Association Test, go-no-go test and contrasting program, and Stroop Test (word and color reading of 112 items during a 2-min period).

### STATISTICAL ANALYSIS

To assess longitudinal differences of cortical thickness in each group, we performed a random field theory using Surfstat package (http://www.math.mcgill.ca/keith/surfstat). In each MSC and placebo group, a longitudinal difference was tested by a general linear model at vertex-wise cortical thickness with controlling for gender and age at baseline characteristics. Multiple comparisons were taken into account for the vertex-wise test using a false discovery rate (FDR; Genovese et al., 2002) correction at a 0.05 level of significance. Next, the correlations of longitudinal changes of cortical thickness and cognitive performance were explored using a multiple, linear regression model in each group. We constructed the regression model accounting with gender and age as independent variables. Initially, we obtained the longitudinal changes of cortical thickness and cognitive scores over 12 months. Then, the multiple, linear regressions between the differences of cortical thickness and cognitive scores were performed in entire vertices and *p* value of 0.05 was assigned to correlation map using an FDR correction. To compare demographic data and changes in subcortical gray matter volumes between groups, the Fisher’s exact test and Mann–Whitney *U* test were used for categorical and continuous variables, respectively. The changes of mean cortical thickness in each group were assessed using Wilcoxon signed rank test. Additionally, Spearman’s correlation analysis was used to evaluate the relationship between the changes in subcortical gray matter volumes and cognitive performance. Statistical analyses were performed using commercially available software (SPSS, version 18.0), and a two-tailed *p* < 0.05 was considered significant.

## RESULTS

### DEMOGRAPHIC CHARACTERISTICS

Baseline and follow-up brain MRI data were available for 11 patients in the MSC group and 15 patients in the placebo group. The demographic characteristics are shown in **Table 1**. There were no significant differences in age at baseline, age at disease onset, gender ratio, disease duration, UMSARS score, Mini-Mental Status Examination (MMSE) score, MoCA score, or education level between the groups.

**Table 1:** Baseline characteristics of the study subjects.

Characteristics	Placebo	MSC	*p* value
No. of subjects	15	11	
Age (year)	55.8 ± 6.3	56.7 ± 8.9	0.77
Sex (female, %)	5 ± 33.3	5 ± 45.5	0.69
Disease duration (months)	33.0 ± 16.7	41.0 ± 13.5	0.25
Total UMSARS score	38.6 ± 6.3	39.2 ± 8.9	0.84
MMSE	27.3 ± 2.5	27.1 ± 2.3	0.88
MoCA	24.1 ± 3.9	24.7 ± 2.9	0.66
Education duration (year)	11.8 ± 4.7	11.7 ± 5.2	0.98
Cortical thickness (mm)	3.20 ± 0.09	3.13 ± 0.12	0.21

*Data are mean ± SD. MSC, mesenchymal stem cells; UMSARS, Unified Multiple System Atrophy Rating Scale; MMSE, Mini-Mental Status Examination; MoCA, Montreal Cognitive Assessment.*

### CHANGES IN SUBCORTICAL DEEP GRAY MATTER VOLUMES AND RELATED COGNITIVE PERFORMANCE

The changes in subcortical deep gray matter volumes assessed by a computerized segmentation procedure are shown in **Table 2**. The volumes of deep gray matter including the caudate, putamen, pallidum, and thalamus were decreased at day 360 relative to baseline in both groups; however, the changes subcortical volumes did not differ significantly between the placebo and MSC groups. In a correlation analysis, the changes in subcortical gray matter volumes did not show a significant correlation with the changes of MoCA score or cognitive performance of specific subdomains in both groups.

**Table 2:** Changes in subcortical deep gray matter volumes between the placebo and MSC groups.

	Placebo (mm^3^)	MSC (mm^3^)	*p* value
Left caudate nucleus	169.87 ± 357.07	175.2 ± 194.7	0.88
Right caudate nucleus	132.32 ± 216.76	117.96 ± 85.17	0.94
Left putamen	378.31 ± 216.43	359.57 ± 227.03	0.99
Right putamen	378.20 ± 210.94	430.04 ± 272.34	0.87
Left pallidum	77.28 ± 105.65	127.31 ± 114.61	0.28
Right pallidum	123.93 ± 151.04	120.90 ± 54.06	0.70
Left thalamus	404.35 ± 237.57	364.51 ± 106.99	0.64
Right thalamus	322.58 ± 241.15	362.63 ± 124.63	0.59

*Data are mean ± SD. MSC, mesenchymal stem cells.*

### CHANGES IN CORTICAL THICKNESS AND RELATED COGNITIVE PERFORMANCE

**FIGURE 1:**
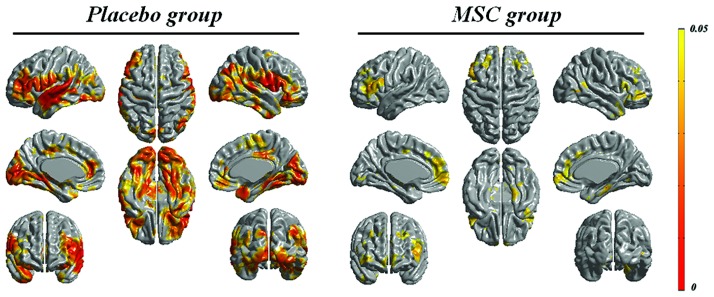
**Longitudinal changes in cortical thickness in both groups.** The areas of cortical thinning in the placebo group during the follow-up period were more extensive, including the frontal, temporal, and parietal areas, whereas cortical thinning over time in the mesenchymal stem cell (MSC) group was less extensive.

The baseline mean cortical thickness in the placebo and MSC groups was 3.20 ± 0.09 and 3.13 ± 0.12 mm, respectively. The change of mean cortical thickness during the follow-up period was 0.097 mm in the placebo group (*p* = 0.005) and 0.069 mm in the MSC group (*p* = 0.012). In comparison to changes in cortical thickness during the follow-up period, the areas of cortical thinning in the placebo group at day 360 compared with the baseline were more extensive, including the frontal, temporal, and parietal areas, whereas cortical thinning in the MSC group during the follow-up period was less extensive and localized mainly to the frontal area (**Figure 1**).

**FIGURE 2:**
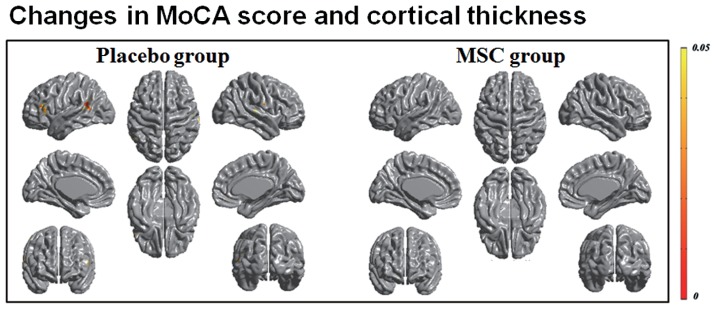
**Correlation analysis of longitudinal changes in cortical thinning and general cognition.** In patients with multiple system atrophy (MSA) receiving placebo, declines in MoCA scores and during the follow-up period were significantly correlated with cortical thinning of the frontal and posterior temporal areas. However, the mesenchymal stem cell (MSC) group had no significant clusters in which the changes in MoCA scores were significantly correlated with the changes in cortical thickness.

**FIGURE 3:**
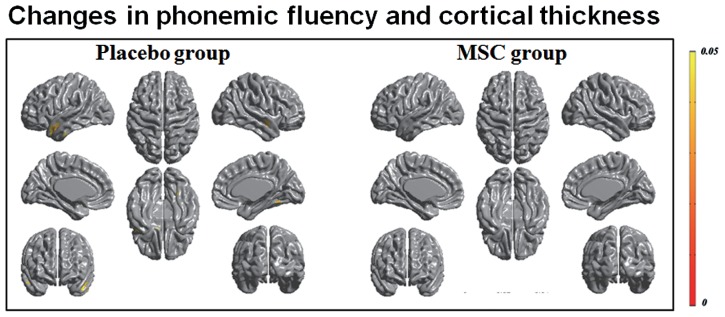
**Correlation analysis of longitudinal changes in cortical thinning and cognitive subdomain scores.** Decrease in phonemic fluency was significantly correlated with cortical thinning in the left anterior temporal area in the placebo group; however, no significant correlations were observed in the mesenchymal stem cell (MSC) group.

Both baseline and follow-up neuropsychological data were available for 10 patients in the MSC group and 15 patients in the placebo group, and the changes in cognitive performance of specific subdomains in each group were reported previously (Lee et al., 2012b). Briefly, the placebo group exhibited a significantly worsening performance in general cognition, forward digit span, naming, visuospatial function, visual and verbal memory, and frontal executive function, whereas the MSC group showed no significant deterioration of cognitive performance, except in Stroop color score. In a correlation analysis of longitudinal changes in cortical thinning and general cognition, the placebo group had significant clusters in which the changes in MoCA scores were significantly and positively correlated with the changes in cortical thickness in the left prefrontal and superior temporal areas (**Figure 2**). In contrast, the MSC group showed no significant correlations between longitudinal changes in MoCA score and cortical thickness. In analyzing the correlations of changes in cortical thickness and each cognitive subdomain performance, only the change in phonemic fluency was significantly and positively correlated with cortical thinning in the left anterior temporal area in the placebo group. In contrast, the MSC group had no significant clusters in which changes in cortical thickness were significantly correlated with change in cognitive subdomain scores (**Figure 3**).

## DISCUSSION

The present study demonstrated that cortical thinning over time, as a primary disease process, is closely associated with cognitive decline in patients with MSA over time. Furthermore, MSC treatment in patients with MSA may modulate longitudinal changes in cortical thickness and its related decline in cognitive performance, suggesting that MSC may have a future therapeutic potential for primary cognitive disorders.

Although dementia is one of the exclusion criteria for the diagnosis of MSA (Gilman et al., 1999), there is evidence that a large portion of MSA patients exhibit a wider range of cognitive dysfunctions than bas been considered (Burk et al., 2006; Kawai et al., 2008; Chang et al., 2009; Brown et al., 2010). With regard to the anatomical substrate of cognitive dysfunction, pathological changes in the striatum and cerebellum can influence cognitive performance in MSA because the cerebellum as well as the striatum contributes to cognitive tasks of frontal executive and language functions and visuospatial performance (Burk et al., 2006; Kawai et al., 2008). In addition, recent pathological and imaging studies have demonstrated widespread cortical involvement in MSA pathology, which may function as a key contributor to cognitive dysfunctions in MSA. Papp and Lantos (1994) reported selective GCI pathology in the primary sensorimotor, supplementary motor, and anterior cingulate cortices in MSA patients. Konagaya et al. (1999) reported a mildly demented MSA patient with prominent frontal atrophy and pronounced GCI distribution in the motor area of the frontal lobe and the parietal lobe. In a voxel-based morphometric study, Brenneis et al. (2006) reported reduced gray matter density in the orbitofrontal, dorsolateral, and medial frontal, parietal, and insular areas, and Lyoo et al. (2008) showed that frontal hypometabolism occurred in the early stages of MSA-cerebellar type. These data suggest that cortical pathology may act as a key contributor to cognitive dysfunction in MSA.

In the present study, we demonstrated longitudinal changes in cortical thickness and its correlation with cognitive performance. In patients with MSA receiving placebo treatment, the areas of cortical thinning during a 1-year follow-up period were extensively widespread throughout the whole cortical area, maximally involving the frontal and temporal areas. These areas show good accordance with the decline in cognitive subdomains of attention, memory, visuospatial function, and executive function in MSA patients receiving placebo. Interestingly, changes in general cognition during follow-up assessed by MoCA were significantly correlated with cortical thinning of the frontal and posterior temporal areas in MSA patients. Furthermore, in analyzing the correlation of changes in specific cognitive subdomains and cortical thinning, decline in phonemic fluency during the follow-up period was significantly correlated with cortical thinning of the anterior temporal area in MSA patients. On the other hand, the changes in subcortical gray matter volumes did not show a significant correlation with the changes of general cognition or cognitive performance of specific subdomains. Therefore, the present data suggest that cortical thinning in MSA would be associated with the primary disease process rather than secondary changes due to degeneration of cerebello-cortical projections or striatal deafferentation, and cortical thinning in these areas are responsible for decline in cognitive performance.

As shown in a previous study indicating that MSC treatment led to significant attenuation of the decrease in cerebral glucose metabolism and gray matter density over time (Lee et al., 2012b), the present study demonstrated that MSC treatment markedly curtailed areas of cortical thinning over time relative to placebo treatment, thus providing another imaging evidence for the neuroprotective effects of MSC with higher statistical power. In addition, in contrast to the placebo group, longitudinal changes in cortical thickness did not show significant correlations with changes in general cognitive performance or specific subdomain performance in MSA patients receiving MSC treatment. On the other hand, longitudinal changes in subcortical deep gray matter volumes did not differ significantly between the placebo and MSC groups. According to our randomized trial, MSC seemed to have neuroprotective effects in patients with MSA-cerebellar type, thus leading to a change in the natural clinical course. Similarly, we postulate that MSC treatment may influence or modulate natural progression of cortical MSA pathology, which may lead to less profound cortical thinning and less decline in cognitive performance over time. As a result, a significant correlation between over-time cortical thinning and cognitive decline is no more evident in MSA patients receiving MSC treatment.

Several studies demonstrated that MSCs can modulate neuroprotective properties in animal models of Alzheimer’s disease (AD). Kim et al. (2012) reported that transplantation of human MSCs in AD transgenic mice enhanced Aβ clearance through augmentation of neprilysin expression in microglia. Recently, Lee et al. (2012a) described that a chemoattractive factor secreted from MSCs may play a critical role in recruitment of alternative microglia into the AD brain and neprilysin derived from the alternative microglia may lead to a reduction in Aβ deposition and memory impairment in AD mice. Furthermore, we very recently demonstrated the modulatory effect of MSCs on autophagy, a vital pathway for degradation of abnormal and aggregated proteins in AD models, showing that MSC treatment significantly enhanced autophagolysosome formation and clearance of Aβ in Aβ-treated cellular and AD transgenic mice with an increased neuronal survival against Aβ toxicity (Shin et al., 2014). Taken together, it can be inferred that the present data may extend clinical application of MSC in primary dementia, such as AD; however, a large amount of cumulative evidence is required to perform a clinical trial.

Some limitations of the present study must to be considered. First, the sample size of the present study was too small to draw a solid conclusion. Second, we recruited patients with MSA-cerebellar type only, because like Japanese MSA patients, MSA-cerebellar type seems to be more prevalent in Korean patients relative to MSA-cerebellar type (Watanabe et al., 2002; Seo et al., 2010). Therefore, our findings may not apply to patients with MSA-Parkinsonian type, and a further study is required to validate our findings. Finally, the present study did not contain the pathological data, and thus, our results could not provide direct evidence that MSC may modulate cortical MSA pathologies and related cognitive performance. Accordingly, our data should be interpreted with caution until pathological data would be available.

In summary, the present data demonstrated that cortical thinning over time, as a primary disease process, is responsible for cognitive decline in patients with MSA over time, and MSC treatment may modulate longitudinal changes in cortical thickness and cognitive performance, suggesting future therapeutic potential for primary dementias.

## Conflict of Interest Statement

The authors declare that the research was conducted in the absence of any commercial or financial relationships that could be construed as a potential conflict of interest.
